# Deep learning model using cross-sequence learning to identify orbital fractures in radiographs of patients under 20 Years

**DOI:** 10.3389/fbioe.2025.1613417

**Published:** 2025-09-02

**Authors:** Joohui Kim, Seungeun Lee, So Min Ahn, Gayoung Choi, Bo-Kyung Je, Beom Jin Park, Yongwon Cho, Saelin Oh

**Affiliations:** ^1^ Department of Radiology, Anam Hospital, Korea University College of Medicine, Seoul, Republic of Korea; ^2^ Department of Mathematics, Korea University, Seoul, Republic of Korea; ^3^ Department of Ophthalmology, Dongguk University Ilsan Hospital, Goyang, Republic of Korea; ^4^ Department of Ophthalmology, Ansan Hospital, Korea University College of Medicine, Ansan, Republic of Korea; ^5^ Department of Radiology, Ansan Hospital, Korea University College of Medicine, Ansan, Republic of Korea; ^6^ Department of Computer Science and Engineering, Soonchunhyang University, Asan, Republic of Korea

**Keywords:** orbital fractures, artificial intelligence, deep learning, radiography, pediatrics

## Abstract

Orbit fractures under 20 years are a medical emergency requiring urgent surgery with the gold standard modality being high-resolution CT. If radiography could be used to identify patients without fractures, the number of unnecessary CT scans could be reduced. The purpose of this study was to develop and validate a deep learning-based multi-input model with a novel cross-sequence learning method, which outperforms the conventional single-input models, to detect orbital fractures on radiographs of young patients. Development datasets for this retrospective study were acquired from two hospitals (n = 904 and n = 910). The datasets included patients with facial trauma who underwent orbital rim view and CT. The development dataset was split into training, tuning, and internal test sets in 7:1:2 ratios. A radiology resident, pediatric radiologist, and ophthalmic surgeon participated in a two-session observer study examining an internal test set, with or without model assistance. The area under the receiver operating characteristic curve (AUROC), sensitivity, specificity, positive predictive value (PPV), negative predictive value (NPV), and 95% confidence intervals (CIs) were obtained. Our proposed model detected orbital fractures with an AUROC of 0.802. The sensitivity, specificity, PPV, and NPV of the model achieved 65.8, 86.5, 70.9, and 83.5%, respectively. With model assistance, all values for orbital fracture detection improved for the ophthalmic surgeon, with a statistically significant difference in specificity (P < 0.001). For the radiology resident, specificity exhibited significant improvement with model assistance (P < 0.001). Our proposed model was able to identify orbital fractures on radiographs, reducing unnecessary CT scans and radiation exposure.

## 1 Introduction

Orbital fractures typically occur due to blunt force trauma, with the relatively thin structures of the orbital floor and medial wall making them more prone to fracture (Gerber et al., 2013). In young pediatric patients, the presence of relatively greater bone elasticity may be associated with “trapdoor” fractures; in these cases, in which the extraocular muscles become entrapped, urgent surgery to prevent permanent muscle damage is required (Joseph and Glavas, 2011). The gold standard modality to detect orbital fractures is thin-sliced high-resolution computed tomography (CT), which provides detailed images of the facial bones (Caranci et al., 2012). Although frequently used to detect orbital fractures, orbital radiographs present a relatively high false–negative rate, ranging (9–28) % (Iinuma et al., 1993).

Deep learning through artificial intelligence (AI) is rapidly advancing, and the medical field is no exception. Deep learning-based fracture detections in various locations, including the shoulder (Uysal et al., 2021), scaphoid (Ozkaya et al., 2022), ribs (Zhou et al., 2020), spine (Murata et al., 2020), and hip joint (Cheng et al., 2019), can achieve high accuracy, with sensitivity and specificity both reaching 91%. Advances in imagery analysis have demonstrated that computer models can assist, and even outperform humans in detecting features of radiographs (Soffer et al., 2019). These models use deep convolutional neural networks (DCNNs), which enable computers to learn features and data patterns that are not readily visible to the human eye. DCNN applications are increasingly used for disease detection and segmentation in medical image analysis. Building on these advances, the development of transformer architectures has further expanded the capabilities of medical image analysis. In particular, Vision Transformers (ViTs) have emerged as powerful alternatives to traditional DCNNs by processing image patches and capturing long-range dependencies through self-attention mechanisms (Dosovitskiy et al., 2020).

Recently, multi-input learning for medical images has gained increased interest. Multi-input models are designed to simultaneously analyze different data formats, such as different imaging modalities or resolutions. However, due to cost and/or time constraints, the simultaneous acquiring of different types of data is not always feasible. To address these limitations, some studies have focused on improving the performance of individual image data using cropping techniques. A two-stage network approach has been proposed (Park et al., 2019; Cho et al., 2021), in which the model first learns from small random patches of the original input images, and then performs transfer learning with whole images. However, this method requires two separate steps, and considerable training time.

This study assumed that DCNNsour multi-input ViT architecture combined with a novel cross-sequence learning technique could assist physicians in identifying orbital fractures and improve patient outcomes. Therefore, we developed and validated a straightforward deep learning-based multi-input model with cross-sequence learning to detect orbital fractures in the plain radiographs of patients under 20 years.

## 2 Materials and methods

### 2.1 Dataset

This retrospective study was approved by the Institutional Review Boards of Korea University Anam Hospital (IRB no. 2022AN0214) and Korea University Ansan Hospital (IRB no. 2022AS0130), and the requirement for informed consent was waived.

**FIGURE 1:**
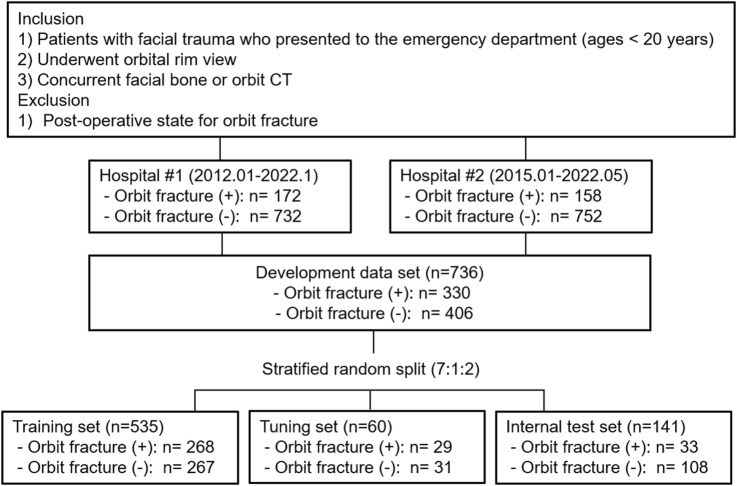
Overview of datasets used in this study.

Orbit radiographs in DICOM format were collected from hospital #1 (January 2012 − January 2022), and from hospital #2 (January 2015 − May 2022). The inclusion criteria for the institution’s computerized medical databases were: 1) patients younger than 20 years who had facial trauma and presented to the emergency department, and 2) patients who underwent an orbital rim view and concurrent facial bone or orbit CT. Orbital rim view is an AP view of the orbit where the orbital rim aligned horizontal to the detector and the central ray enters the head at a 10–15°. CT images were obtained with various CT scanners at two institutions (hospital #1: Somatom Definition AS and Somatom Definition Flash, Siemens Healthcare, Forchheim, Germany, or Brilliance 64, Philips Healthcare, Amsterdam, Netherlands and hospital #2: Aquilion ONE, Toshiba, Minato, Japan or Revolution, GE Healthcare, Chicago, IL, USA). The most frequently used scanning parameters were as follows: tube voltage, 120 kVp; effective tube current, 250 mAs; section thickness, 2 mm; pitch, 0.8; rotation time, 1.0 s; and collimation, 128 × 0.6 mm.Patients with postoperative state for orbital fracture were excluded. The reference standard for orbital fracture diagnosis is facial bone or orbit CT. Two radiologists (S.O. and G.C., with 9 and 7 years experience, respectively, of pediatric imaging interpretation) were blinded to the clinical information, and reviewed the CT scans independently. They evaluated the presence of orbital fractures, and recorded their locations. The locations were classified as superior, medial, lateral, floor, or multiple. The reviewers resolved any disagreements by consensus. The development dataset was randomly split into training, tuning, and internal test sets in approximate ratios of 7:1:2 at the patient level, in a stratified manner based on the labels. Additionally, we employed stratified 10-fold cross validation. To improve training quality and balance, patients without orbital fractures were randomly selected for the training and tuning sets. Figure 1 presents the details of the data set.

### 2.2 Classification model

This study proposes a new learning technique termed cross-sequence learning for multi-input image classification models. The approach involves two parallel ViTs without specially designed feature fusion encoders, except for one concatenation layer. A cropped image based on the region of interest (ROI) was generated for each image. Each cropped image was generated by manually isolating only the head region based on the ROI and the orbital area, excluding the neck and any background elements. Since the cropping process only excludes regions irrelevant to diagnosis, the label of each cropped image remains consistent with its corresponding original image. Input image diversity was increased by matching different input types based on their lowest cosine similarity. To compare the performance of multi-input models with cross-sequence learning, single- and multi-input models without cross-sequence learning were designed, respectively.

**FIGURE 2:**
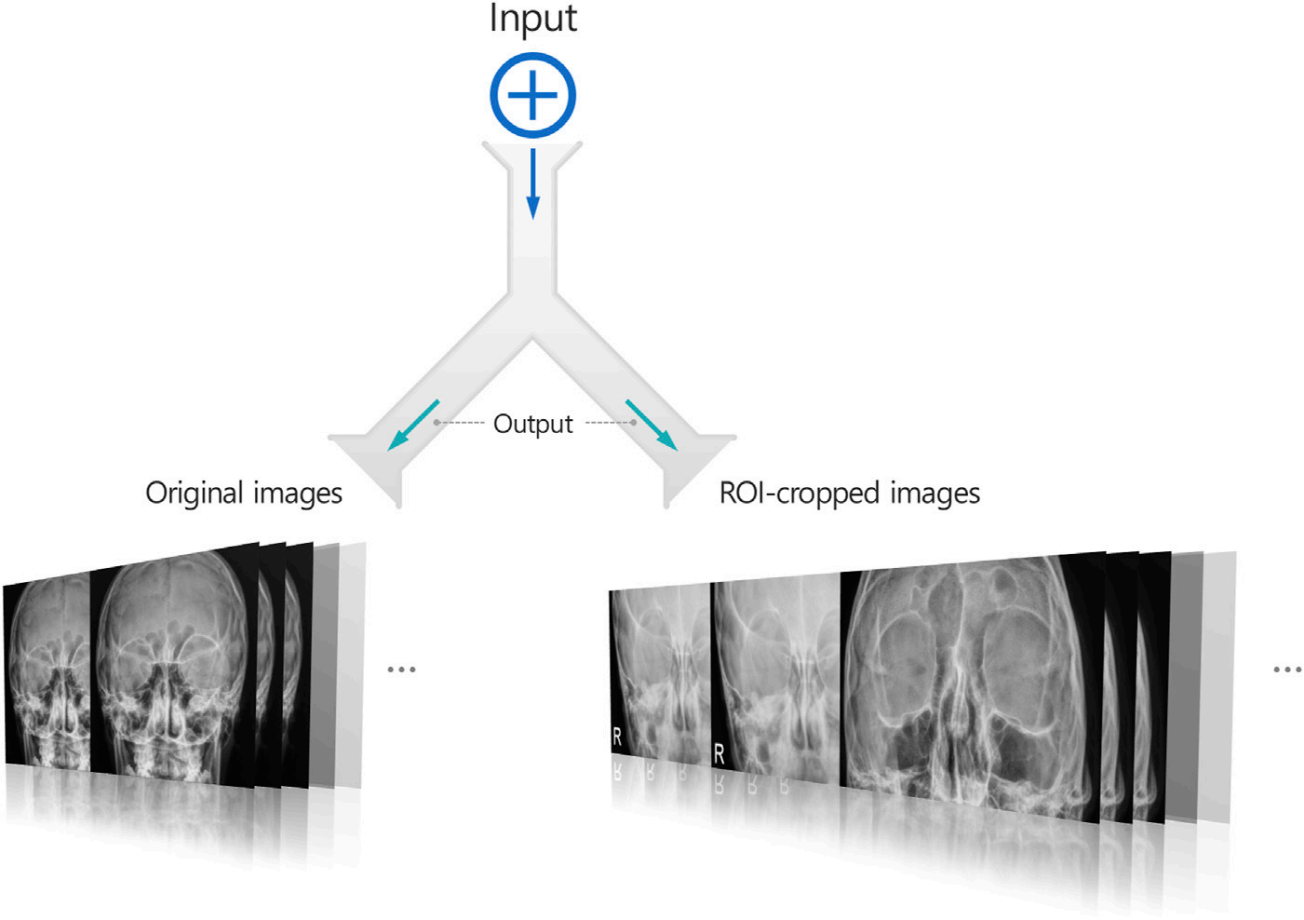
A multi-input image classification model generating two images for each input image: The original image and a cropped image based on the ROI.

**FIGURE 3:**
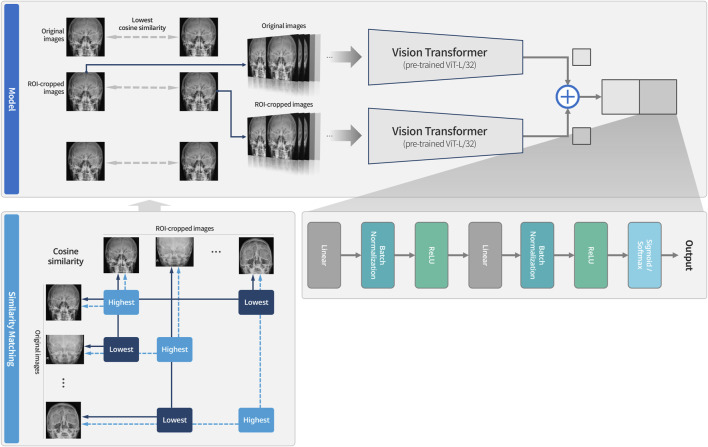
System overview of multi-input models with cross-sequence learning for orbital fracture detection. The dotted line indicates cases that could not be matched due to having the highest values, while the solid line represents cases that were matched because they had the lowest values. After the similarity matching process, the paired images are used as input to the model. Training is then conducted using the Vision Transformer for original images and the Vision Transformer for ROI-cropped images, respectively.

**FIGURE 4:**
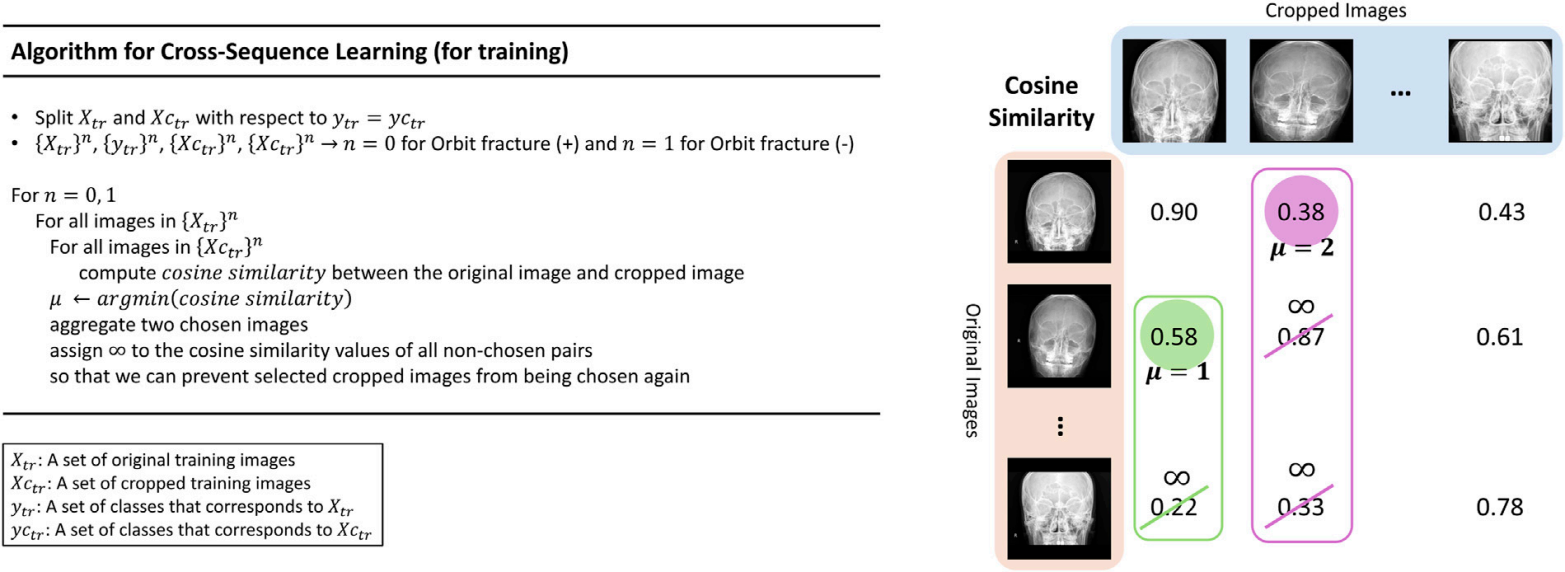
Algorithmic pipeline of cross-sequence learning.

Multi-input image classification models with cross-sequence learning use two images for each input: the original, and the cropped image based on the ROI (Figure 2). In a multi-input model with two ViTs in parallel, the first ViT processes the original image, while the second ViT processes the cropped image as the input. The features extracted from both ViTs are concatenated, and passed through the fully connected layers for binary classification. Cross-sequence learning consists of two steps (Figures 3, 4): Step 1 determines the pair of images by selecting the index of the cropped image having the lowest cosine similarity with each original image. In this step, we ensure that the matched images share the same given label to prevent mixing data from different classes during training, which may cause label confusion. It is also noteworthy that we calculated the cosine similarity based on the raw pixel values of each image. In step 2) prohibits the selected cropped images from being chosen again to increase input diversity. The matched original and cropped images were then combined and used as inputs for the multi-input classification model. Both the ViTs processed the original and the ROI-cropped images. The extracted features were concatenated sequentially for the final classification. Cross-sequence learning was not necessary for the validation and testing processes. The original and ROI-cropped images with the same indices were used as inputs for the multi-input model. More algorithmic details can be found in the Appendix.

### 2.3 Clinical validation

Clinical validation was performed using internal validation test. Three readers participated in a two-session review of the orbital rim view: a pediatric radiologist with 9 years of experience (reader 1), an ophthalmic surgeon with 9 years of experience (reader 2), and a radiology resident with 2 years of experience (reader 3). Anonymized original DICOM files (excluding age and sex) were provided. Readers were informed that the study included young patients with facial trauma. Radiographs only were obtained in the first session. The second session was held 1 month after the first session. Readers were provided with model assistance, and the review order of the patients was altered. High-probability areas were highlighted as the most likely fracture sites in the original image. In both sessions, the readers recorded the final reading of each patient’s orbital fracture (with or without AI results) on a five-point scale (1 = definitely normal; 2 = probably normal; 3 = indeterminate; 4 = probable fracture; 5 = definite fracture).

### 2.4 Statistical analysis

Fracture detection accuracy was evaluated using the area under the receiver operating characteristic curve (AUROC), sensitivity, specificity, positive predictive value (PPV), and negative predictive value (NPV). Optimal cutoff values for sensitivity and specificity were assessed using the Youden index (Youden, 1950), which is the point on the ROC that maximizes both sensitivity and specificity.

For human readers, AUROC values were obtained using five-point diagnostic confidence levels, and then dichotomized into normal (scores (1–3)) and fractured (scores 4 and 5) for binary diagnosis. Sensitivity, specificity, PPV, and NPV were obtained from the confusion matrices. The DeLong method (DeLong et al., 1988) was used to compare individual AUROC values, and McNemar’s test was used to compare the sensitivity and specificity values. A P value of less than 0.05 was considered statistically significant. MedCalc version 22.007 (MedCalc Software BVBA) was used for all statistical analyses.

## 3 Results

### 3.1 Patients

This study included 172 patients with orbital fracture, and 732 without, from Hospital #1; and 158 with, and 752 without, from Hospital #2. Of the 1,814 included radiographs (330 [18%] with orbital fracture, and 1,484 [82%] without), the most common site of orbital fractures was the orbital floor (35%, 116/330), followed by multiple fractures (32%, 104/330), and medial wall fractures (28%, 91/330). A total of 206 patients (62%) underwent surgery for orbital fracture repair, with a median interval of 4 days (interquartile range, 2–7 days) between diagnosis and surgery. The most common surgical site was multiple orbital walls (40%, 82/206), with 70% (81/116) of the patients undergoing surgery for orbital floor fractures (Table 1).

**TABLE 1:** Baseline clinical characteristics of the patients with orbital fractures.

Characteristic	Value
Sex	​
Male	291 (88)
Female	39 (12)
Age at orbital fracture diagnosis, years^a^	16.1 ± 4.1
Fracture site	​
Floor	116 (35)
Medial	91 (28)
Superior	16 (5)
Lateral	3 (1)
Multiple	104 (32)
Surgery	206 (62)
Floor^b^	81 (70)
Medial^b^	39 (43)
Superior^b^	3 (19)
Lateral^b^	1 (33)
Multiple^b^	82 (79)
Interval between orbital fracture diagnosis and surgery, days^c^	4 (2–7)

Unless otherwise indicated, data are numbers of patients with percentages in parentheses.

^a^ Data are presented as means ± standard deviation.

^b^ Percentages of patients who underwent surgery within the fracture site are indicated in parentheses.

^c^ Data are presented as medians with interquartile ranges in parentheses.

### 3.2 Standalone performance of the deep learning model

**FIGURE 5:**
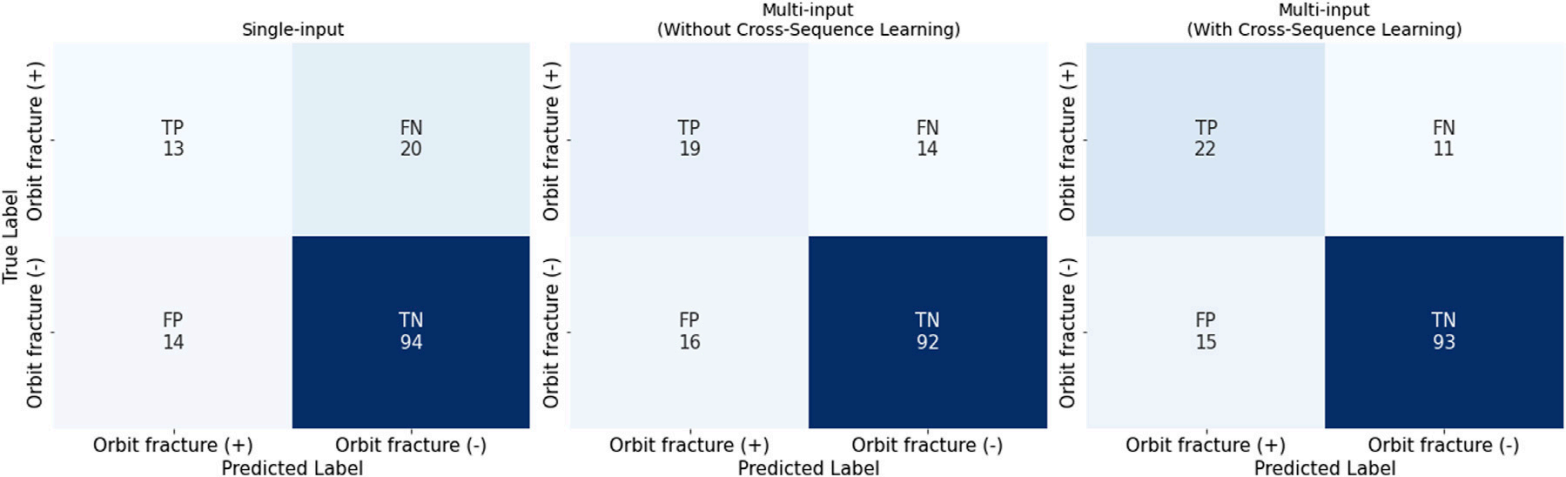
Confusion Matrices for the Three Settings: single-input, multi-input without/with cross-sequence learning. It is noteworthy that we have conducted stratified 10-fold cross validation.

The single-input model created with ViT achieved an AUROC of 0.670 and a specificity of 0.871 (Table 2; Figure 5), and a low sensitivity (0.387), with PPV of 0.600 and an NPV of 0.740. The multi-input model without cross-sequence learning showed improved sensitivity (0.580) and AUROC (0.800), with slightly improved PPV and NPV (0.666 and 0.803, respectively).

**TABLE 2:** Model performance to detect orbital fractures according to input and cross-sequence learning.

	AUROC	Sensitivity	Specificity	PPV	NPV	F1-score
Single-input model	0.670	0.387	0.871	0.600	0.740	0.471
Multi-input model						
Without cross-sequence learning	0.800	0.580	0.855	0.666	0.803	0.620
With cross-sequence learning	0.802	0.658	0.865	0.709	0.835	0.683

AUROC, area under the receiver operating characteristic curve; PPV, positive predictive value; NPV, negative predictive value.

**FIGURE 6:**
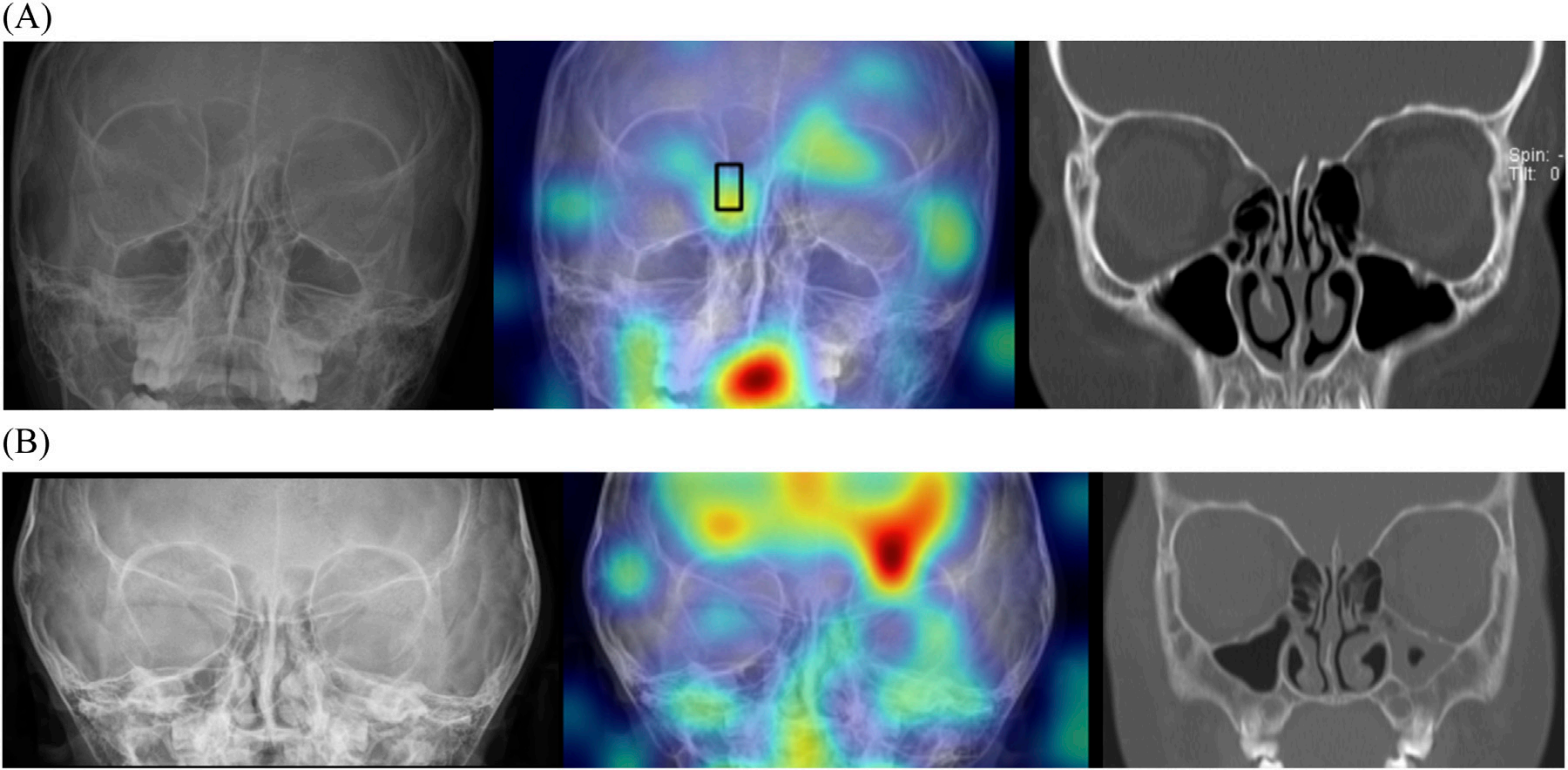
Localizing fracture sites using gradient-weighted class activation mapping. **(A)** Representative true-positive case of a 10-year-old boy with orbital fracture. Right medial wall fracture of the orbit was correctly localized by the model (black box). **(B)** Representative true-negative case of a 3-year-old girl without an orbital fracture. This model does not identify any fractures; therefore, no bounding box is offered.

Multi-input image classification models using cross-sequence learning matched the original images with the cropped images of other patients to increase learning diversity. By setting the number of epochs to 400, all values exhibited slight improvement (AUROC, 0.802; sensitivity, 0.658; specificity, 0.865; PPV, 0.709; NPV, 0.835). Figure 6 presents the representative true–positive and true–negative cases from the internal test set.

### 3.3 Observer performance with and without deep learning model assistance

**FIGURE 7:**
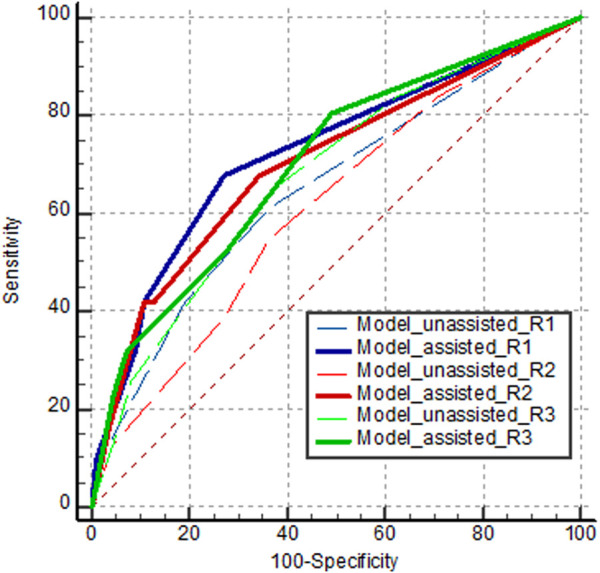
AUROC curves of human readers diagnosing orbital fracture on the internal test set. Dashed and solid lines indicate the first session without model assistance and the second session with model assistance, respectively.

Table 3 and Figure 7 show the diagnostic performance of human readers in the internal test set with, and without, model assistance. In the first session, reader AUROCs ranged (0.611–0.676). The sensitivity and specificity of the observers ranged (38.7–64.5) %, and (63.6–80.9) %, respectively. The range of PPV of the readers was relatively low at (28.6–38.2) %, compared to that of NPV at (80.8–86.4) %.

**TABLE 3:** Diagnostic performance of human readers in the external test set with and without the model’s assistance.

	R1		R2		R3		Model-unassisted vs model-assisted (*p* value)		
	Model-unassisted	Model-assisted	Model-unassisted	Model-assisted	Model-unassisted	Model-assisted	R1	R2	R3
AUROC	0.650 (0.565–0.728)	0.724 (0.643–0.796)	0.611 (0.525–0.692)	0.700 (0.617–0.774)	0.676 (0.592–0.752)	0.701 (0.618–0.775)	0.32	0.25	0.43
Sensitivity, %	41.9 (24.6–60.9)	32.3 (16.7–51.4)	38.7 (21.9–57.8)	41.9 (24.6–60.9)	64.5 (45.4–80.8)	51.6 (33.1–69.9)	0.63	1.00	0.22
Specificity, %	80.9 (72.3–87.8)	90.9 (83.9–95.6)	72.7 (63.4–80.8)	89.1 (81.7–94.2)	63.6 (53.9–72.6)	72.7 (63.4–80.8)	0.06	<0.01	<0.01
PPV, %	38.2 (26.0–52.1)	50.0 (31.4–68.6)	28.6 (18.9–40.7)	52.0 (35.5–68.1)	33.3 (25.9–41.7)	34.8 (25.2–45.7)			
NPV, %	83.2 (78.3–87.1)	82.6 (78.8–85.9)	80.8 (75.7–85.1)	84.5 (80.0–88.1)	86.4 (79.5–91.3)	84.2 (78.5–88.7)			

Numbers in parentheses are 95% confidence interval.

AUROC, area under the receiver operating characteristic curve*;* PPV, positive predictive value*;* NPV, negative predictive value.

In the second session with model assistance, some performance parameters improved for some readers. All readers exhibited higher specificities (improvements of (9.1–16.4) %), with statistical significance for two readers (P < 0.001 for reader 2, and P < 0.001 for reader 3). Compared to the first session, all readers showed AUROC improvements (reader 1: 0.075; 95% confidence interval [CI], (−0.073–0.222); P = 0.323; reader 2: 0.089; 95% CI, (−0.061–0.240); P = 0.245; reader 3: 0.025; 95% CI, (−0.038–0.088); P = 0.432), without statistical significance. Readers 1 and 2 yielded significant improvements in PPV at (11.8 and 23.4) %, respectively, whereas reader 3 exhibited minimal improvement in PPV (1.5%).

## 4 Discussion

This study developed and validated deep learning-based models to differentiate normal and fractured orbits on plain radiographs. With an AUROC of 0.802, the results of our multi-input model with cross-sequence learning suggest that deep learning methods, such as those analyzing orbital fractures, can detect bone fractures that are difficult for human readers to evaluate. This study also demonstrated the feasibility and clinical validity of a deep learning algorithm to diagnose orbital fractures on plain radiographs. Several recent studies have applied convolutional neural network training models to detect different types of fractures in radiographs (Yang et al., 2020; Gan et al., 2019). Their performance in long bone or limb joint fractures achieved excellent accuracy of approximately 90% (Lindsey et al., 2018; Chung et al., 2018; Pelka et al., 2018; Kuo et al., 2022). Few studies have investigated the pediatric population (Choi et al., 2020; Hayashi et al., 2022; Zech et al., 2023), and only one study involved fractures other than those in the long bones (Choi et al., 2022). To the best of our knowledge, this is the first study to develop a deep neural network model to detect orbital fractures on plain radiographs in a population under 20 years. Li et al. reported an AUROC of 0.958 to detect orbital fractures using orbital CT scans in an adult population (Li et al., 2020).

Orbital fractures have various presentations and clinical severities. The anatomical complexity of the orbital and intraorbital structures also creates confusion (Caranci et al., 2012). Radiography has a sensitivity of (64–78) % for fractures. Currently, radiographic examination of the orbits is rarely performed (Iinuma et al., 1994). CT is considered the imaging modality of choice to evaluate orbital trauma (Kubal, 2008). Three-dimensional reformation is a useful tool for guide treatment (Rhea et al., 1999), though it requires prolonged hospitalization and radiation exposure. Pediatric orbital fractures differ from those in adults, with diplopia, muscle entrapment, and trapdoor configuration fractures being more common in children (Lane et al., 2007). Urgent surgery is indicated to prevent soft tissue scarring and its long-term sequelae (Bansagi and Meyer, 2000). Our study demonstrated a short interval between orbital fracture diagnosis and surgery in clinical practice. Additionally, orbital fractures were present in only 18% of the patients who underwent CT at our institutions, suggesting unnecessary radiation exposure. If radiography could be used to identify patients without fractures, the number of unnecessary CT scans and radiation could be reduced.

Deep learning-based models can learn features and data patterns that are invisible to the human eye. Training deep learning-based models for medical image analysis requires access to substantial, high-quality, and well-annotated datasets. A multi-input approach was proposed to improve the quality of the dataset. This approach can be applied to various tasks that include image classification, segmentation, and restoration (Duarte et al., 2018; Chen et al., 2022; Yang et al., 2022). For example, a multi-modal fusion method (Chen et al., 2022) combines the analysis of images, graphs, and genomic data, whereas a multi-resolution fusion model (Yang et al., 2022) examines the same object from various perspectives. However, due to cost and/or time constraints, most multi-input models that simultaneously obtain different types of data are infeasible. Furthermore, most multi-input models typically involve specialized preceding architectures for feature fusion. Previous studies (Duarte et al., 2018; Chen et al., 2022) introduced Siamese networks and Kronecker products for multi-input image analysis. However, when compared to single models, these models tend to be more complex. Other studies have proposed a two-stage network approach (Park et al., 2019; Cho et al., 2021), in which the model first learns from small random patches of the original input images, and then performs transfer learning with whole images. Nevertheless, this method requires two separate steps and considerable training time, as sufficient patches are required to achieve a meaningful performance.

This study developed a multi-input ViT architecture with a similarity-matching mechanism to identify the original and ROI-cropped images with the lowest cosine similarity for multi-input image classification. Our experiments validate the effectiveness of the proposed framework using two different datasets. We also considered that similarity matching could reduce unintended errors, and provide important information regarding ROIs. This matching can quantitatively and qualitatively enhance the performance of multi-input models. Future work will incorporate graph neural networks to create a trainable similarity function rather than a cosine similarity function, and use multiple parallel architectures to generate cropped images of various sizes from the original images.

This study has limitations. Our models were trained on large datasets from two academic institutions. Hence, to improve the accuracy and generalization of models, further assessment of large datasets from other centers is required. Despite balancing the training and tuning sets, our model exhibited a relatively low sensitivity, possibly due to the small number of orbital fractures included in the study. However, the improved sensitivity of multi-input models with cross-sequence learning indicates that our proposed models can detect non-visible fractures. Although the sensitivity of our model is relatively low, it remains comparable to that of expert clinicians. We anticipate that, in emergency settings, our model could still provide more reliable results than those obtained by individuals who are less experienced in interpreting orbital radiographs. Finally, as not all patients with facial trauma undergo both orbital radiography and CT, indication bias is another potential limitation, considering that the patients included in the training set had a high likelihood of orbit fractures and underwent CT.

In this study, our multi-input ViTs with cross-sequence learning method were developed to identify orbital fractures using radiography. Sensitivity and specificity at encouraging levels were achieved, suggesting that the models can detect bone fractures that are difficult for human readers to evaluate. This study also determined that the multi-input models with cross-sequence learning could improve the detection of fractures that are not readily visible to physicians. This enhanced diagnostic capacity can help solve medical problems with high monetary or quality-of-life costs, and improve fracture care.

## Data Availability

The raw data supporting the conclusions of this article will be made available by the authors, without undue reservation.

## Appendix

**Configuration of Vision Transformer (ViT):** We use the pre-trained ViT-L/32 as our backbone network, which is a large variant of Vision Transformer with a patch size of 32×32 with an embedding dimension of 1,024, 24 transformer layers, and 16 attention heads. The model is further fine-tuned on our custom dataset, and two parallel ViTs are trained independently.

**Feature Representations:** We extract the full sequence of patch token embeddings from the final transformer layer of ViT-L/32, excluding the CLS token. Especially, we extract features from the last normalization layer of ViT, before the final attention block. An input resolution of 384×384 results in 12×12 patches, each with a 1024-dimensional embedding. This eventually yields a total feature shape of 144×1024 per image. Instead of relying on the CLS token, we use the full set of patch embeddings to retrain richer spatial features for downstream classification tasks.

Concatenating two outputs of ViTs: We concatenate the outputs along the patch dimension, resulting in a combined shape of 288×1024 . This is then flattened to a 1D vector before being passed to the fully connected layers.

Final Classifier Architecture, Final Classifier Architecture: The concatenated feature vector is fed into a fully connected (FC) classifier with the following architecture.

(1) FC Layer ( $R^{294912}$ → $R^{1000}$ ), followed by Batch Normalization, Dropout with a ratio of 0.3, and ReLU
(2) FC Layer ( $R^{1000}$ → $R^{100}$ ), followed by Batch Normalization, Dropout with a ratio of 0.3, and ReLU
(3) FC Layer ( $R^{100}$ → $R^{1}$ ), followed by Sigmoid activation for binary classification

**Training details:** The goal of our experiments is to reduce the binary cross entropy loss, using a batch size of 64 and an initial learning rate of 0.001. We use the Adam optimizer and apply learning rate decay via a LambdaLR scheduler, which multiplies the learning rate by 0.95 after every epoch. The training is conducted for 100 epochs. All experiments are performed on a GPU server running Ubuntu 20.04 with CUDA 11.2 and three 24 GB Titan RTX graphics cards. All models are implemented using PyTorch 1.8.0.

**Data Preprocessing:** For all images, including cropped ones, we apply standard scaling and then resize images to 384×384 using the Lanczos interpolation over 8×8 neighborhoods.

**Algorithmic Details of Cosine Similarity-based Matching:** The entire pipeline is illustrated in Figure 4, along with the algorithm and corresponding graphics.
